# Mitochondria: Emerging Consequential in Sickle Cell Disease

**DOI:** 10.3390/jcm12030765

**Published:** 2023-01-18

**Authors:** Mohammad S. Akhter, Hassan A. Hamali, Hina Rashid, Gasim Dobie, Aymen M. Madkhali, Abdullah A. Mobarki, Johannes Oldenburg, Arijit Biswas

**Affiliations:** 1 Department of Medical Laboratory Technology, College of Applied Medical Sciences, Jazan University, Jizan 45142, Saudi Arabia; 2 Department of Pharmacology and Toxicology, College of Pharmacy, Jazan University, Jizan 45142, Saudi Arabia; 3 Institute of Experimental Haematology and Transfusion Medicine, University Clinic Bonn, 53127 Bonn, Germany

**Keywords:** sickle cell disease, mitochondria, mitochondrial retention, ROS, mtDNA

## Abstract

Advanced mitochondrial multi-omics indicate a multi-facet involvement of mitochondria in the physiology of the cell, changing the perception of mitochondria from being just the energy-generating organelles to organelles that highly influence cell structure, function, signaling, and cell fate. This sets mitochondrial dysfunction in the centerstage of numerous acquired and genetic diseases. Sickle cell disease is also being increasingly associated with mitochondrial anomalies and the pathophysiology of sickle cell disease finds mitochondria at crucial intersections in the pathological cascade. Altered mitophagy, increased ROS, and mitochondrial DNA all contribute to the condition and its severity. Such mitochondrial aberrations lead to consequent mitochondrial retention in red blood cells in sickle cell diseases, increased oxidation in the cellular environment, inflammation, worsened vaso-occlusive crisis, etc. There are increasing studies indicating mitochondrial significance in sickle cell disease, consequently providing an opportunity to target it for improving the outcomes of treatment. Identification of the impaired mitochondrial attributes in sickle cell disease and their modulation by therapeutic interventions can impart a better management of the disease. This review aims to describe the mitochondria in the perspective of sicke cell disease so as to provide the reader an overview of the emerging mitochondrial stance in sickle cell disease.

## 1. Introduction

**Figure 1:**
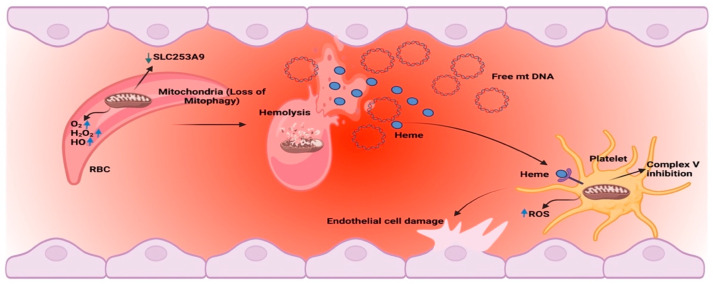
The mitochondrial dynamics and energetics are impaired in SCD, and are indicated by mitochondrial retention in RBCs, increased oxidative stress, and free circulating mtDNA.

The mitochondria are emerging as highly influential organelles in the health and disease of eukaryotic cells. They are primarily implicated in cellular bioenergetics and regulation of critical cellular processes besides being focal points of intracellular organelle interactions and vital responsive sensing systems in the cell [1,2]. Mitochondria exhibit a heteroplasmic genome and require about 1500 proteins from two genomes to maintain their normal physiology [3,4]. Stressors and/or mutations in the mitochondrial or cellular genome can impair the normal functions of the mitochondria, leading to dysfunctions or impaired dynamics [3,4]. Such mitochondrial dysfunctions and impaired dynamics individually or in coaction with other pre-existing pathologies are increasingly being recognized as an endorsement of various diseases [5]. The consequential role of mitochondria is emerging in the hematopoietic cell homeostasis at various critical points, such as oxidative phosphorylation (OXPHOS), regulation of reactive oxygen species (ROS), caspase activation, heme synthesis, etc. [6,7]. Bioenergetic dysfunction, altered dynamics, or pathogenic mutations in mitochondrial DNA (mtDNA) have been identified to be pivotal in numerous hematological disorders [6,8,9]. Sickle cell disease (SCD) and its variants are a group of autosomal recessive hematological inherited disorders that present diverse clinical manifestations with multisystemic organ damage [7]. SCD is a significant cause of morbidity and mortality, particularly in people of African and Mediterranean ancestry, and considerably lowers the quality of life in such patients when compared to patients of other chronic diseases [10,11]. SCD pathophysiology involves molecular, cellular, and biophysical events that are induced by a single gene mutation causing polymerization of hemoglobin S (HbS) (β^Glu6Val^), which leads to the formation of sickle cells under the conditions of de-oxygenation. The generation of the rigid sickle cells is associated with impaired biorheology and increased adhesion-mediated vaso-occlusion, elevated hemolysis-mediated endothelial dysfunction, and activated inflammation [1,12]. Increasing studies identify mitochondria as cardinal players in the pathophysiology of SCD, where mtDNA, bioenergetic dysfunction, mitochondrial hyperpolarization, oxidant generation, and transporter proteins may steer the course of the disease [7,13,14] (Figure 1). Besides variations in nuclear DNA, polymorphic variations in mtDNA are reported to be marked in patients with SCD and may influence the disease severity [15]. This manuscript aims to discuss various aspects of mitochondrial involvement in sickle cell disease and mitochondria as a therapeutic target in SCD.

## 2. Mitochondrial Retention

The normal RBC maturation in mammals demonstrates an elaborate process that exhibits extensive cellular remodeling, leading to terminal enucleation followed by mitochondrial and other organelle loss, and a shift to anaerobic glycolysis [16]. This is to optimize the critical functions of the RBC, which are hemoglobin production, oxygen transport, and deformability. The mitochondria play a vital role during erythropoiesis, where they are reprogrammed to propel the differentiation process and are key to nuclear clearance, before being eliminated by mitophagy [16]. Mitochondrial dynamics (MtDy) are reported to be the significant regulatory point in this transformation [17]. The mitochondrial extrusion is suggested to help the mammal erythrocytes to limit ROS production and survive better in high-sugar and high-heme conditions since aerobic energy production in eukaryotic cells inevitably produces ROS as by-products, which can lead to mtDNA mutations, dysfunction, and, therefore, apoptosis [18,19]. Hence, the mitophagy can be perceived as a cytoprotective mechanism preventing cell death [18]. A salient feature of the RBC in SCD is the retention of the mitochondria owing to compromised mitophagy pathways. Studies with SCD patients and murine models of SCD have demonstrated mitochondrial retention to be a feature of RBC, with increased R1 reticulocytes in the peripheral blood of SCD patients compared to control subjects [20]. The percentage of mitochondrial retention in RBCs directly correlates with the absolute number of reticulocytes, which in turn links mitochondrial retention with hemolysis in SCD patients and an elevated oxygen consumption rate in SCD RBCs [20]. Hong et al., 2019 [21] reported a selenium-deficient diet to cause an increase in mitochondrial retention in mature RBCs, lower hemoglobin levels, and elevated RBC oxygen consumption in humanized SCD mice. The timely elimination of the mitochondria in RBC is regulated by mitophagy genes and defects in such genes leads to decreased RBC survival and severe anemia arising out of defects in mitochondrial clearance. *Atg7* is an essential autophagy gene and mice lacking this gene develop severe anemia as *Atg7−/−* erythrocytes tend to cumulate impaired mitochondria with altered membrane potential, causing cell death. It is revealed by proteomic analysis of erythrocyte ghosts that other cellular degradation mechanisms are induced in absence of autophagy. Non-essential autophagy genes *Ulk1* and Nix are also involved in the mitophagy during erythrocyte maturation and are upregulated during terminal erythroid differentiation [18]. The absence of *Ulk1* and Nix in mice is associated with mild nonlethal anemia [22,23]. Treatment of SCD mice with RN-1 was observed to have more than two-fold expression of essential mitophagy genes, including the erythroid-specific mitophagy gene *Atg7,* when comparison was made to untreated SCD mice, indicating a role of mitophagy genes in the development of SCD [24].

Recent studies by Martino et al., 2021 [25] reported abnormal (40%) retention of mitochondria in SCD patients with decreased levels of mitophagy-inducer proteins PINK1 and NIX and elevated levels of HSP90 chaperone in their red cells.

Stress erythropoiesis in SCD is also emerging as a major contributor of erythrocyte mitochondrial retention. Gallivan et al., 2022 reported reticulocytosis from stress erythropoiesis to be a major source of erythrocyte mitochondrial retention, oxygen consumption, and reactive oxygen species in the SCD mouse model [26]. Rai et al., 2020 reported in their studies with mice that angiotensin signaling had a significant role in stress erythropoiesis in SCD but resulted in the retention of dysfunctional mitochondria in erythrocytes that in turn generated excessive reactive oxygen species [27]. Ramasamy et al., 2022 reported an abnormal presence of mitochondria in circulating red blood cells associated with stress erythropoiesis in SCD patients [28].

Moras et al., 2022 reported for the first time a significant role for outer mitochondrial membrane protein voltage-dependent anion channel-1 (VDAC1) in human erythroblast terminal differentiation, regulating mitochondria clearance of the in-human terminal erythropoiesis. They demonstrated mitochondria clearance to start at the transition from the basophilic to polychromatic erythroblast and that VDAC1 downregulation induced mitochondrial retention. They also showed VDAC1 to be involved in phagophore’s membrane recruitment regulating selective mitophagy of functional mitochondria from human erythroblasts [29].

It was reported by Moriconi et al., 2022 that RBCs from SCD patients had functionally active mitochondria that could elicit a type 1 interferon response. It was confirmed by flow cytometry, electron microscopy, and proteomic analysis that the mitochondria in mature RBCs were metabolically competent, which increased oxidative stress, in turn facilitating and/or intensifying SCD complications. They reported the coculture of mitochondria-positive RBCs with neutrophil-produced type 1 interferons, which are known to increase RBC alloimmunization rates, thereby suggesting that incongruous mitochondrial retention in RBCs may play a significant role in SCD complications and be an RBC alloimmunization risk factor [30].

The numerous pathways leading to mitochondrial retention in SCD can be targeted by pharmacological agents and explored as a therapeutic approach towards SCD.

## 3. Oxidative Stress

Oxidative stress is an important element in the pathophysiology of SCD. Chronic and systemic oxidative stress in SCD can result via repetitive polymerization and depolymerization of hemoglobin (Hb) in RBC, activated leukocytes, platelets, ECs, and plasma enzymes [31,32]. At subcellular levels, ROS generation by mitochondria is one of the major contributors to elevated oxidative stress in various tissues in SCD. In SCD RBC, free radical production is linked to auto-oxidative unstable HbS, dissociated heme, membrane-bound hemichrome, heme free iron, NADPH oxidase, and excessive adenosine triphosphate utilization concurrent with an increased rate of HbS polymerization and mitochondrial retention. The retention of mitochondria in SCD is suggested as one of the significant sources of elevated intracellular ROS in SCD RBC, with its interplay with NADPH oxidase [20,33]. Upregulated NADPH oxidase catalytic subunits are characteristic features of SCD RBC, indicating an oxidative environment [34]. The mitochondria feature the highest levels of antioxidants in the cell, are critical in maintaining the cellular redox status, and act as ROS and redox sinks, in turn limiting NADPH oxidase activity [33]. However, they are also a major source of ROS generation, especially through electron leakage from complexes I (primary) and III of the electron transport chain (ETC). A total of 0.2–2% of the electrons in the ETC escape the normal fate and directly leak out of the ETC, generating superoxide or hydrogen peroxide by interacting with oxygen [35]. These ROS originating from mitochondria can play in the vicious cycle where they activate NADPH oxidase, which in turn stimulates mitochondrial ROS (mtROS) generation, causing aggravation of intracellular OS [33]. Downregulation of expression levels of antioxidant levels in mitochondria in SCD may also contribute to elevated ROS production in SCD RBC [36].

Increased platelet mtROS are observed in SCD, which may be triggered by bioenergetic alterations in mitochondria, leading to an increased mtROS production [37]. Platelets in SCD have diminished mitochondrial respiration, higher mitochondrial membrane potentials, and produce higher levels of H2O2. There is an indicated mitochondrial complex V dysfunction in SDC platelets that leads to mitochondrial hyperpolarization and elevated H2O2 generation [38].

An efficient molecule that is necessary for maintaining redox balance in cells is glutathione (GSH). Mitochondria need sufficient levels of GSH for biosynthetic reactions and for maintaining redox balance. Recently, SLC25A39 has been identified as a mitochondrial membrane carrier that regulates GSH transport into mitochondria; however, its function is unknown. A loss of SLC25A39 was associated with decreased mitochondrial GSH import and levels, with no effect on cytosolic GSH levels. The cells that lacked SLC25A39 and its paralogue SLC25A40 had iron–sulfur clusters containing proteins with impaired activity and stability. Since GSH import by mitochondria is essential for red blood cell development in mice, it may be possible that there is an involvement of fallible GSH import protein in SCD [39].

Mitochondria contribute to the elevated OS in SCD due to an increase in mitochondrial ROS production and mitochondrial damage, resulting due to compromised SOD2 synthesis [36]. SOD2 is a significant antioxidant enzyme that is synthesized in the cytoplasm and is localized to the mitochondrial matrix and primarily dismutates the superoxide anion that is generated by respiratory chain enzymes. SOD2 expression is regulated by ROS and RNS, while its enzymatic activity is regulated post-translationally. Phosphorylation at serine 106 by Cdk1 increases the enzymatic activity of SOD2 and increases cell survival, while acetylation of lysine 122 and lysine 68 by a loss of SIRT3 leads to inhibition of enzymatic activity [40,41]. Peroxynitrite and superoxide radicals have an antagonistic mechanism of regulating SOD2 expression, implicating a dysregulated mitochondrial antioxidant response in SCD. Elevated levels of peroxynitrite can lead to nitration at tyrosine 34 causing enzymatic inhibition, while superoxide radicals upregulate SOD2 expression by activating the redox-sensitive transcription factors NF-κB and Nrf2 [42]. Additionally, there is an impaired SOD2 activation by superoxide radicals in SCD. It has been noted that SCD patients have reduced peripheral blood SOD2 mRNA levels compared to controls, suggesting a reduced expression of SOD2 [43].

Lately, another enzymatic antioxidant Glutathione peroxidase 4 (GPX4) has gained importance as a significant modulator of oxidative stress in red blood cells. GPX4 is an antioxidant defense enzyme found in mitochondria as well as other subcellular organelles and is distinguished since it is the only enzyme that prevents adverse lipid peroxidation in vivo by reducing lipid peroxides to the respective alcohols, hence stabilizing oxidation products of unsaturated fatty acids [44]. An intensive proteome analysis of the RBCs has established the presence of GPX4 protein in these cells [45]. The plenitude of GPX4 correlates with lipid-anchored proteins, strongly indicating the role of GPX4 in maintaining homeostasis in RBCs. GPX4 has a critical role in scavenging ROS and lipid hydroperoxides in the erythroid lineage, and a loss of GPX4 causes erythroid precursor cell death, leading to anemia. It has been shown in studies with mice that deletion of *GPX4* in hematopoietic cells leads to the development of anemia and that GPX4 is essential for preventing receptor-interacting protein 3 (*Rip3*)-dependent necroptosis in erythroid precursor cells. It has been observed that deletion of Rip*3* normalizes reticulocyte maturation and prevents anemia, but the ROS accumulation and lipid peroxidation in *GPX4*-deficient cells are highly elevated, further endorsing a key role of GPX4 in the homeostasis of ROS and lipid hydroperoxides in erythroid precursors [46]. It was reported by Stolwijk et al., 2021 that during the storage of RBCs, the lower concentration and activity of GPX4 were associated with increased oxidative damage by the reactions of phospholipid-OOH, consequently increasing hemolysis [47]. The GPX4 genes are negatively associated with SCD [48]. A significant role of GPX4 has been observed in mitophagy as well, and a GPX4 deficiency in reticulocytes leads to their failure to mature fully. Mitophagy is dependent on lipid oxidation, which is controlled by GPX4, under normal physiological conditions. An uncontrolled auto-oxidation is observed in case of a lack of GPX4, causing disrupted autophagosome maturation and hence perturbing mitophagy [49].

## 4. Hemolysis

Hemolysis is a basal component of SCD and contributes significantly to its pathophysiology. The products of hemolysis include increased circulating free heme and plasma hemoglobin, enhanced ROS generation, decompartmentalized hemoglobin, asymmetric dimethylarginine, arginase 1, and adenine nucleotides which contribute to complications and disease severity [50]. The circulating free heme is a potent oxidative molecule that acts as an erythrocytic danger-associated molecular pattern (eDAMP) molecule which can activate Toll-like receptor-4 (TLR4) of the innate immune system, causing oxidant production, inflammation, and vascular injury [51,52]. Heme and cell-free Hb are reported to disrupt mitochondrial bioenergetic function in different cell types [53]. Higher oxidized forms of hemoglobin (HbS, βV6E, and HbE βE26K) from SCD patients cause endothelial dysfunction via a loss of respiratory chain complex activities in isolated endothelial mitochondria, while prolonged incubation with ferryl Hb (HbFe4^+^) induced bioenergetic reprogramming, including a higher degree of uncoupled respiration and glycolytic rate [54]. In a study to evaluate the role of Hb redox reactions on the alveolar type (AT) 1 cell, (HbFe2^+^-) and ferric Hb (HbFe3^+^-) were observed to induce upregulation of heme oxygenase (HO-1) expression in the mitochondria. A significant decrease in basal mitochondrial respiration and cellular glycolytic flux was observed with exposure to HbFe3^+^, indicating a loss of mitochondrial bioenergetic function. HbFe4^+^ caused a loss of mitochondrial transmembrane potential (Δψm), leading to significant mitochondrial depolarization or compromised mitochondrial respiration [53]. HbFe4^+^ also leads to complex formation with band 3 and RBC membrane proteins [54]. Band 3 proteins belong to the anion exchange family (AE 0–3), which are present in the membranes of all cells and cellular organelles, such as the Golgi body, mitochondria, and nuclei. Band 3 proteins maintain cell volume and osmotic homeostasis, aging of RBC, HCO3-/Cl- exchange, IgG binding, cellular removal, and the maintenance of the structural integrity of cells [55]. There is tissue variance in the expression of the mitochondrial protein; however, the protein may have a role in mitochondrial anion transport [56]. SCD features and accelerated aging of band 3 possibly impair the mitochondrial membrane [55].

Mitochondrial activation of platelets is also triggered by hemolysis and mitochondria evolve to be involved at multilevels. Plasma Hb or free heme released as a product of hemolysis stimulates platelet thrombotic activation via inhibition of complex V of the platelet mitochondrial electron transport chain, causing an increase in mtROS production, consequently stimulating platelet activation. However, it is reported that cell-free heme is more potent in activating platelets as compared to Hb, besides inducing granule release. Platelet granule secretion is stimulated by heme and regulated by mitochondrial oxidants. Heme was observed to induce the release of eight granule factors, TSP1, CXCL7, FGF basic, TGFβ, IL-1β, PDGF-B, angiostatin, and kininogen, under the influence of mtROS. Heme-dependent TLR4 activation was also found to be heme-dependent, which inhibited mitochondrial complex V and induced mtROS production through both MyD88-dependent and independent pathways. Heme mediates activation of TLR4 signaling that stimulates downstream serine kinase Akt phosphorylation, a protein that binds and phosphorylates various mitochondrial proteins (including α and β subunits of complex V) and regulates their function, causing inhibition of complex V activity [57]. The inhibition, in turn, increases mtROS causing platelet activation and granule secretion. AKT-dependent phosphorylation of ATP synthase also regulates mitochondrial bioenergetics by increasing substrate availability and regulating the catalytic/energy-transducing capacity of mitochondria [58].

## 5. mtDNA Variation

Variations in mtDNA are a determining factor in disease since a wide array of common clinical phenotypes are associated with mtDNA haplogroups. The haplogroups also influence predisposition to metabolic, degenerative, infectious, autoimmune diseases, and various cancers [48,59]. mtDNA variation can influence cellular and organismal phenotypes through retrograde signaling to the nucleus as one of the possible mechanisms. Variations and mutations in coding and control regions of mtDNA have significant roles in various inherited and acquired diseases [60]. SCD also exhibits variations in the mtDNA control region. The control region is the most polymorphic region of the human mtDNA genome, having the highest polymorphism in hypervariable regions with an average nucleotide diversity of 1.7% [61]. Sequencing of the mitochondrial control region in SCD from nucleotide position (np) 16,024 to np 210, which encompassed the Hyper Variable Segment I and most of the Hyper Variable Segment II, revealed 99 polymorphic sites comprising 10 transversions, 1 deletion, and 1 insertion (Table 1) [15]. Unusual C-phosphate-G (CpG) and non-CpG methylation within the human mtDNA control region, ie., the D-loop, has an unexpected prevalence in non-CpG moieties. Both the CpG and non-CpG methylated sites are located within the promoter region of the heavy strand (P_H_), within the conserved sequence blocks (CSBI-III), both being highly conserved sequences at the 5′-end of the D-loop, suggesting a key role of D-loop methylation in modulating the replication or transcription of mtDNA. The variability arising due to polymorphisms can lead to the creation/suppression of CpGs. Increased methylation levels were observed in human blood and HeLa cells when compared to fibroblasts and osteosarcoma cells [51,62]. These observations indicate a possible convoluted role of methylation and variance in mtDNA, which may be extended to SCD. Ahmed et al. (2020) reported an increase in the frequency of heteroplasmic variants in SCD in comparison to ethnic-matched healthy populations. A genotype-dependent progressive increase in heteroplasmic burden was observed (HbAS < HbSβ^+^ thalassemia < HbSC < HbSS and HbSβ^0^), which was associated with increasing phenotypic severity [63]. Mitochondrial heteroplasmy varies within tissues or cells even in the same individual. All these findings concomitant with chronic inflammation in SCD strongly suggest increased rates of mtDNA mutations in SCD. It still cannot be concluded whether the variants are a cause or effect of the sickle inflammatory pathology; however, it can be inferred that mtDNA heteroplasmic burden may be deemed as a key biomarker of SCD severity.

## 6. Circulating Mitochondrial DNA

SCD patients feature disproportionate elevation in levels of cell-free mitochondrial DNA (cf-mtDNA) over cell-free nuclear DNA (cf-nDNA) when compared to controls. The elevated levels of cf-mtDNA likely arise from the retained mitochondria in SCD RBC. The size of cf-mtDNA (median size ∼131 bp) is smaller compared to cf-nDNA (median size ∼168 bp), cell mtDNA (cmtDNA) (median size ∼442 bp), and cell nuclear DNA (cnDNA) (median size, ∼437 bp). cf-mtDNA is disproportionately methylated in SCD and shows robust hypomethylation in SCD patients during crises compared with their respective steady states. The cf-mtDNA acts as eDAMP and triggers the formation of neutrophil extracellular traps (NETs) via a cytosolic pathway involving serine/threonine TANK-binding kinase 1 and activation of the cGAS-STING pathway in the process. cf-mtDNA is suggested to be the major NET trigger in SCD, whereas the heme/hemin content might contribute secondarily [6]. The cf-mtDNA act as potent pro-inflammatory molecules by triggering NETs and due to hypomethylation, as levels of DNA methylation are associated with inflammation.

## 7. Therapeutic Targeting

**Figure 2:**
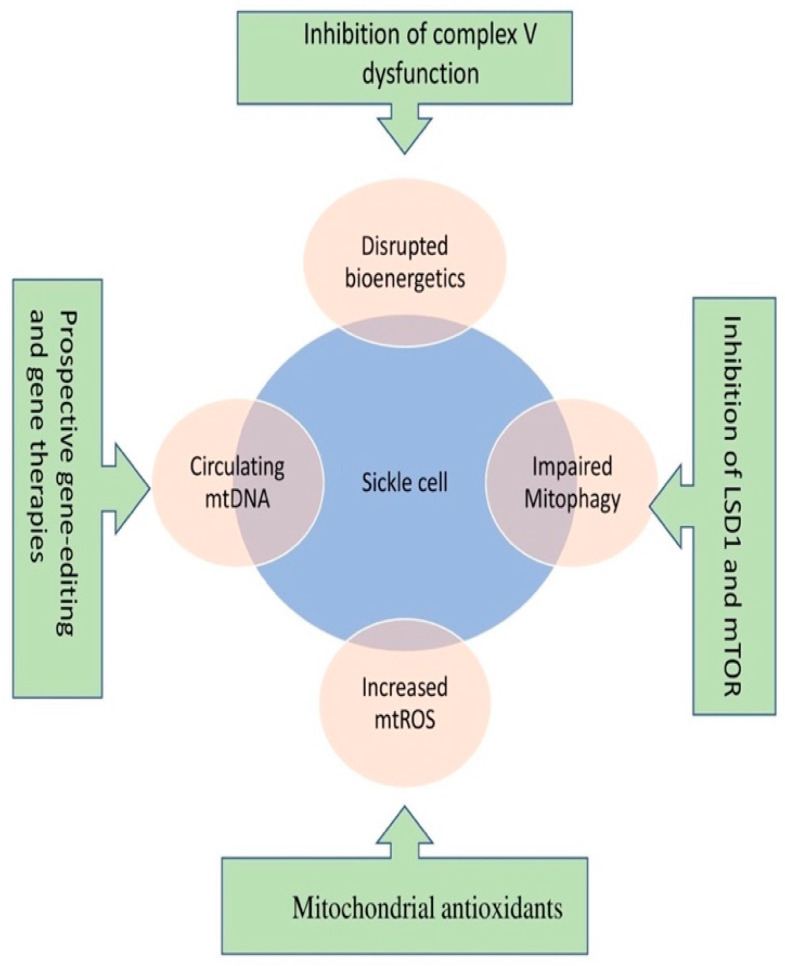
An all-inclusive approach to modulate bioenergetics, dynamics, and genome of mitochondria is of therapeutic importance in improving the treatment outcome in SCD.

The emerging position of mitochondria as a significant organelle in SCD pathophysiology entails it as a propitious pharmacological target to limit the processes that involve mitochondria (Figure 2).

It can be an organelle of specific interest in precision medicine to identify the severity and course of the disease (Table 2). Impaired mitophagy is a characteristic feature of SCD that leads to mitochondrial retention and pharmacological intervention to induce mitophagy can be a new therapeutic approach in SCD. Jagadeeswaran et al., 2017 [24] reported that two preclinical candidate drugs, the LSD1 inhibitor, RN-1, and the mammalian target of rapamycin (mTOR) inhibitor, sirolimus, both reduced mitochondrial-retaining RBCs in the SCD mouse model. They also observed a two-fold induction in the expression of major mitophagy genes including the Atg7 gene in the bone marrow cells obtained from SCD mice treated with RN-1. RN-1 mediates epigenetic upregulation of pro-mitophagy genes and causes mitochondrial clearance in SCD [34,39]. Sirolimus is known to inhibit mTOR and promote autophagy in mitochondria in human cell hybrid lines carrying pathological mtDNA mutations. Active mTORC1 kinase signaling represents repressed macroautophagy and has phosphorylated downstream targets, including the ribosomal S6 protein. However, cells treated with mTORC1 inhibitor exhibited a signal for phospho-S6, implying inhibition of mTORC1 signaling and enabling the induction of macroautophagy [64]. mTOR inhibition also improved anemia and reduced organ damage in a murine model of SCD [14]. The treatment with RN-1 sirolimus was also associated with a decrease in ROS in SCD [24]. A deficiency in selenium was also observed to increase mitochondrial retention in SCD mice, which was attenuated by feeding a selenium-adequate diet to mice. This can be of therapeutic significance where mitochondrial dynamics are impaired due to nutritional deficiencies of trace minerals. Lowered mitochondrial complex V activity in platelets in SCD potentiates mitochondrial membrane potential and leads to increased oxidant production from the electron transport chain. This dysfunction is augmented by hemolysis and causes platelet activation and is associated with low arginine bioavailability.

A significant therapeutic approach is arginine supplimentation. It is a conditionally essential amino acid and a necessary substrate for nitric oxide (NO) synthesis. NO is a potent vasodilator necessary for maintaining vascular homeostasis; low NO bioavailability promotes sickle cell vasculopathy. SCD presents decreased arginine bioavailability correlating with pulmonary hypertension risk, early mortality, and pain severity, predicting the need for pediatric hospitalization [65]. Altered mitochondrial function in SCD arises partly due to low nitric oxide bioavailability. Treatment with arginine was observed to be associated with decreased disease severity. Various trials were and are being conducted to evaluate the efficacy of arginine in SCD. In a trial by Morris et al., 2020, 12 pediatric patients presenting vaso-occlusive pain episodes at the emergency department presentation had significantly decreased complex V activity compared to a steady-state cohort. The complex V activity significantly increased at discharge in subjects from IV arginine-dosing schemes. Arginine treatment significantly increased mitochondrial activity and decreased protein-carbonyl levels, indicating a decrease in oxidative stress [66].

Another trial by Reyes et al., 2022 also supported IV arginine administration for the treatment of SCD patients hospitalized with vaso-occlusive episodes. They evaluated 108 patients aged 3–21 and observed that IV arginine therapy was well tolerated with no unexpected severe adverse effects, and severe adverse effects/adverse effects rates were similar across study arms [65].

Oral administration of arginine in SCD patients aged 5–17 years has also yielded positive results. In their trial, Onalo et al., 2022, randomized sixty-six SCD children into arginine versus placebo groups, and multiple indices of cardiopulmonary hemodynamics and mean N-terminal pro-B-type brain natriuretic peptide were evaluated after oral arginine therapy. Oral arginine therapy was observed to improve cardiopulmonary hemodynamics during sickle cell disease vaso-occlusive pain and acute chest syndrome [67].

L-glutamine, an L-α-amino acid, has also emanated as a prospective therapeutic molecule with antioxidant activities in SCD. It has shown beneficial treatment outcomes in patients five years old and older [68]. It is an amino acid with a wide array of roles in the body, spanning from the synthesis of antioxidants, such as reduced glutathione, and the cofactors NAD(H) and NADP(H), as well as nitric oxide. However, it is still not clear how L-glutamine modulates the redox environment in red blood cells in sickle cell disease, and a few therapeutic trials in sickle cell disease are being carried out [69]. Glutamine, being the most abundant amino acid in the body, is considered a non-essential amino acid; however, a high RBC turnover due to hemolysis in SCD increases the demand for glutamine, making it a conditionally essential amino acid in such patients [70]. Glutamine supplementation is reported to alleviate acute complications in SCD patients by acting as a substrate for the synthesis of glutathione and synthesis of NAD(H) and NADP(H). In a multicenter, randomized, placebo-controlled, double-blind phase 3 trial, Niihara et al., 2018 reported a reduction in the pain crisis over 48 weeks in those patients who received oral therapy with L-glutamine than those receiving a placebo, for both adult and pediatric patients [71]. Recently Walter et al., 2022 in their clinical trial reported oral L-glutamine to upregulate apoptotic and autophagy proteins BAX (mitochondrial apoptotic marker) and LC3-II/LC3-I in SCD [72].

**Table 2:** Mitochondrial markers in SCD.

Mitochondrial Feature	Increase/Decrease or Presence/Absence	Consequence	Implication	References
Cell-free DNA	Increased	Triggers NETs formation	Pathological inflammation	[6]
Hypomethylation of mtDNA	Increased	Triggers NETs formation	Increased crises	[7]
Complex V activity	Decreased	Increased ROS	Hemolysis	[13,66]
PINK1	Decreased	Impaired mitophagy	Mitochondrial retention in RBC	[25]
NIX	Decreased	Impaired mitophagy	Mitochondrial retention in RBC	[25]
SOD2^V16A^	Present	Decreases mitochondrial Complex IV activity	Increased tricuspid regurgitant velocity	[36,66]
SOD2 expression	Decreased	Decreased SOD levels	Increased oxidative stress in microenvironment.	[43]

## 8. Conclusions

Impaired mitochondrial functions are a significant attribute of SCD and increasing studies have strongly suggested mitochondria to be involved in the occurrence and severity of SCD. Identification of mitochondrial involvement at various levels in the disease provides a power to make pharmacological inventions at a mitochondrial level to possibly improve the quality and span of life in SCD patients. It is a highly significant field that needs to be explored exhaustively with advanced technologies to find the convolutions of mitochondria with the cell physiology in SCD to find better therapeutic interventions that are yet lacking.

## Figures and Tables

**Table 1:** mtDNA sequence variation and geographical distribution of mtDNA in sickle cell patients.

S. No.	Ethnic Origin	Haplotype ID	Haplogroup	GenBank Accession Number
1	Europe	HT20	T1a1 + 152	MT176208
2	America	HT29	B4	MT176209
3	Africa	HT40	G1a	MT176210
4	Africa	HT40	G1a	MT176211
5	Africa	HT32	L2a1	MT176212
6	Africa	HT32	L2a1	MT176213
7	Africa	HT09	L1b2a	MT176214
8	Africa	HT21	L1b1a + 189	MT176215
9	Africa	HT41	L2a1 + 143	MT176216
10	Europe	HT39	L3e1b2	MT176217
11	Africa	HT10	L2a1	MT176218
12	Africa	HT03	L1c1d	MT176219
13	Africa	HT08	L1c2a1a	MT176220
14	Africa	HT04	L2a1a1	MT176221
15	Africa	HT35	M30c	MT176222
16	America	HT44	U7a	MT176223
17	Africa	HT34	L2a1 + 16189 + (16192)	MT176224
18	Africa	HT13	L3k1	MT176225
19	Africa	HT26	L0a1a2	MT176226
20	Africa	HT25	L0a1a	MT176227

Adapted from Barbanera et al., 2020 [15].

## Data Availability

No new data were created or analyzed in this study. Data sharing is not applicable to this article.
